# Multi-threshold polygenic risk improves hippocampal-based cognitive decline prediction

**DOI:** 10.1186/s13073-026-01722-x

**Published:** 2026-07-17

**Authors:** Mohammed Janahi, Luigi Lorenzini, Neil P. Oxtoby, Frederik Barkhof, Younes Mokrab, Jonathan M. Schott, Andre Altmann, for the Alzheimer’s Disease Neuroimaging Initiative

**Affiliations:** 1 The UCL Hawkes Institute and Department of Medical Physics and Biomedical Engineering, University College London, Gower Street, London, WC1E 6BT UK; 2 Medical and Population Genomics Lab, Research Branch, Sidra Medicine, Doha, Qatar; 3 Department of Radiology and Nuclear Medicine, Amsterdam University Medical Centre, Vrije Universiteit, 1081 BT Amsterdam, The Netherlands; 4 Amsterdam Neuroscience, Brain Imaging, 1081 BT Amsterdam, The Netherlands; 5 UCL Hawkes Institute and Department of Computer Science, University College London, Gower Street, London, WC1E 6BT UK; 6 Centre for Medical Image Computing, Queen Square Institute of Neurology, University College London, London, UK; 7 Department of Genetic Medicine, Weill Cornell Medicine-Qatar, Doha, Qatar; 8 Department of Biomedical Science, College of Health Sciences, Qatar University, Doha, Qatar; 9 Dementia Research Centre (DRC), Queen Square Institute of Neurology, University College London, Gower Street, London, WC1E 6BT UK; 10 Dementia Research Institute, University College London, London, UK

**Keywords:** Polygenic Scores, Normative Modelling, Brain Modelling, Dementia, Neurodegenerative Disorders

## Abstract

**Background:**

Predicting cognitive decline from brain MRI is a central question in neuroscience. Hippocampal volume (HV) is a key cognitive biomarker, and normative models can be augmented with multimodal information. Here we augment normative models with genetic information and show improvements in cognitive decline prediction across multiple experimental setups.

**Methods:**

We improve normative models for HV by integrating multi-threshold polygenic scores (PGS) with demographic and imaging data using Gaussian Process Regression (GPR). Models were trained on 23,997 participants from UK Biobank (UKBB) and validated on 3,000 out-of-sample participants from the Alzheimer’s Disease Neuroimaging Initiative (ADNI) and the European Prevention of Alzheimer's Disease (EPAD) cohorts.

**Results:**

Our genetically-informed models significantly strengthened associations across six experimental designs and 13 key neurocognitive measures, including Mini-Mental State Examination (MMSE), Clinical Dementia Rating (CDR), and Alzheimer’s Disease Assessment Scale (ADAS), while enhancing prediction of future cognitive decline.

**Conclusions:**

Together, these findings underscore the promise of integrating multi-threshold PGS with neuroimaging-based predictive models to improve prognostication and early intervention strategies for neurodegenerative diseases.

**Supplementary Information:**

The online version contains supplementary material available at https://doi.org/10.1186/s13073-026-01722-x.

## Background

Brain imaging provides invaluable insights into the pathophysiology of brain disorders. Over the past decades, various case–control studies have identified multiple disease hot-spots in the brain, notably large-scale meta-analyses by the ENIGMA consortium which established prototypical abnormality maps for dozens of brain disorders [1]. However, the paradigm of case–control studies assumes that all cases express the same disease-related imaging pattern, which is unlikely given the heterogenous nature of most brain disorders. Increasingly, normative modelling is used in neuroimaging research, comparing individual subjects’ brains to the expected appearance in a healthy cohort to identify personalized disease maps [2–4]. As such, normative modelling was shown to be effective in modelling heterogeneous diseases like schizophrenia and attention deficit hyperactivity disorder (ADHD) [2, 5]. Recent work has established normative brain models mapping the entire lifespan based on over 100,000 participants [6]. However, classic normative models typically ignore the effect of genetics. For instance, hippocampal volume (HV) in healthy individuals has been associated with numerous loci [7]; most prominently *TESC*, *MSRB3* and *LEMD3*, in addition to a large number of variants with small effects (i.e. polygenic effect)*.* Moreover, overexpression of *TESC* in mice was found to enhance neurite outgrowth in hippocampal neurons leading to increased HV [8]. Conversely, the association with *MSRB3* may originate from is function of preventing apoptosis leading to HV loss [9]. Janahi et al. demonstrated that normative models can be improved by incorporating polygenic scores (PGS), which capture genetic predisposition to variation in a brain phenotype of interest such as HV, thereby allowing abnormality detection up to three years earlier [10]. Other studies have similarly found significant benefits of genetic adjustment in normative modelling [11, 12].

PGS are typically constructed as a weighted combination of Single Nucleotide Polymorphisms (SNPs) [13], deriving the weights from Genome Wide Association Studies (GWAS)[14]. PGS were calculated to measure genetic risk across a range of clinical phenotypes including coronary artery disease, diabetes, and Alzheimer’s disease (AD) [15]. Furthermore, PGSs were used to estimate long term health trajectories in Schizophrenia, AD and ADHD [16–20].

Linkage Disequilibrium (LD) poses a challenge when creating PGSs. While GWAS estimates independent SNP effects, SNPs are often inherited not individually, but as haplotype blocks; [21] consequently, PGS construction needs to adjust for LD. The standard method for LD adjustment is Clumping and Thresholding (C + T), where SNPs below a certain p-value threshold are selected, followed by clumping neighbouring SNPs from the same LD haplotype, then retaining the highest effect size SNP in each clump [22]. For the remaining SNPs, the effect sizes are then weighted by an individual’s genotype (0,1, or 2 copies of the SNP) and summed to produce the PGS. More advanced methods adjust all GWAS SNP effect estimates, combining all SNPs without clumping or thresholding, assuming either a small (e.g., LD-Pred [23], SBayesR [24]) or a large number of diseasing-causing SNPs (e.g., PRS-CS [25], lassosum [26], SBLUP [27]). However, C + T method remains the most widely used [28], assuming a wide range of P-value thresholds ranging from stringent (P < 5e-8) and selecting SNPs with low standard errors, to lenient cut-offs (P < 0.5) allowing better coverage but less accurately estimated effects. While a single best performing threshold is often selected for downstream analysis, there is mounting evidence that PGSs at different thresholds capture different aspects of the trait and are not simply interchangeable[29–32]. For instance, Stacked Clumping and Thresholding which combines approximately 100,000 PGS using a penalized logistic regression, leads to improved disease prediction performance [33].

**Fig. 1:**
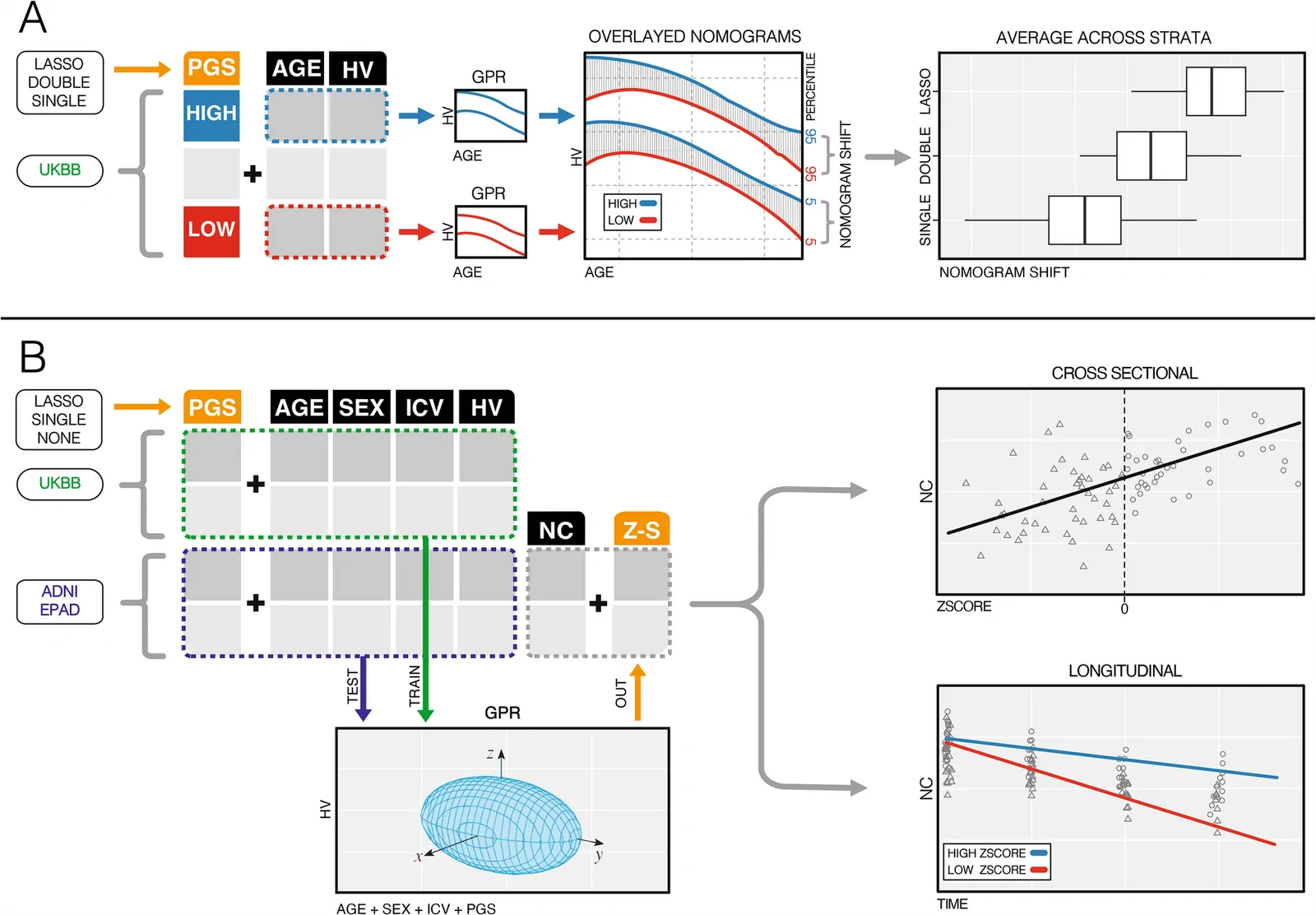
Overview of the study leveraging multi-threshold polygenic scores to improve prediction of cognitive decline. To explore the value of combing multiple *p*-value thresholds of the same C + T based polygenic scores (PGS) in predicting cognitive decline, (**A**) we generate PGS using single, pairs, and 13 combined thresholds (LASSO-PGS) for UK Biobank (UKBB). We then train Gaussian Process Regression (GPR) models on high/low PGS participants (top/bottom 30%) and report the predicted model differences. (**B**) Next, we train UKBB models with no PGS, single threshold PGS, or LASSO-PGS and test them in two out-of-sample, harmonized datasets (ADNI, EPAD) to obtain HV z-scores. We finally correlate the z-scores to multiple neurocognitive measures such as memory and orientation, cross-sectionally and longitudinally

In this study, we explore the value of combining multiple P-value thresholds to improve normative models for HV and its clinical application for prognosis of cognitive decline (Fig. 1A). First, we assess the information in HV PGSs at different thresholds and use machine learning approaches to leverage them. Next, we integrate the combined PGSs into multivariate normative models based on data from UK Biobank (UKBB) [34] and evaluate them in two out-of-sample cohorts across the AD spectrum: the Alzheimer’s Disease Neuroimaging Initiative (ADNI) database [35], which comprises cognitively normal subjects and subjects with mild cognitive impairment and AD, and the European Prevention of Alzheimer's Disease (EPAD) database [36], which comprises cognitively normal participants at risk for dementia (Fig. 1B). Finally, we compare the models to standard in terms of correlations to various neurocognitive measures, including memory and orientation, both cross-sectionally and longitudinally.

## Methods

### Pre-processing

In total, three datasets were used in this study, summarized in Table 1 with detailed imaging and genetic acquisition parameters provided in Additional File 1: Table S1.

**Table 1:** Summary of pre-processed datasets

	UKBB (*n* = 23,997)	EPAD (*n* = 1,755)	ADNI (*n* = 1,245)		
			CN (*n* = 479)	MCI (*n* = 617)	AD (*n* = 149)
Age (mean, SD)	64.4 (7.5)	65.8 (7.1)	71.5 (5.2)	71.0 (6.7)	71.6 (7.2)
Sex					
Male	11,799 (49.2%)	774 (44.1%)	198 (41.3%)	356 (57.7%)	84 (56.4%)
Female	12,198 (50.8%)	981 (55.9%)	281 (58.7%)	261 (42.3%)	65 (43.6%)
HV mm3 (mean/SD)	4043 (398)	3994 (408)	3938 (339)	3679 (401)	3418 (399)
Follow-up					
N		1523	450	589	143
Mean (years)/SD		1.03 (.75)	2.7 (2.3)	2.5 (2.1)	2.2 (1.8)
MMSE					
N		1755	479	617	149
Mean/SD		28.4 (1.8)	29.0 (1.1)	27.9 (1.7)	23.2 (2.1)
CDR-SB					
N		1752	479	617	149
Mean/SD		0.36 (0.74)	0.03 (0.13)	1.4 (0.8)	4.3 (1.5)
ECOG					
N			434	448	116
Mean/SD			1.37 (.32)	1.78 (.53)	1.9 (.64)
ADAS					
N			476	614	146
Mean/SD			8.45 (4.4)	15.3 (6.6)	30.8 (8.7)
MOCA					
N			432	446	119
Mean/SD			26.1 (2.46)	23.59 (3.05)	17.19 (4.7)
RBANS					
N		1745			
Mean/SD		101 (15)			
Flanker					
N		1014			
Mean/SD		8.3 (.8)			
DotCount					
N		1167			
Mean/SD		16.7 (4.6)			

*HV* Hippocampal Volume (after cross-dataset harmonization; Methods), *MMSE* Mini-Mental State Exam, *CDR-SB* Clinical Dementia Rating – Sum of Boxes, *ECOG* Everyday Cognition, *ADAS* Alzheimer's Disease Assessment Scale, *MOCA* Montreal Cognitive Assessment, *RBANS* Repeatable Battery for the Assessment of Neuropsychological Status, *DotCount* Dot Counting

#### UKBB

The UK Biobank (UKBB) is a population cohort study of around 500 k participants from the UK and contains a vast wealth of data including imaging, genetics, and clinical biomarkers [34]. From a total of 501,172 (272,747 female) participants (application number 65299), we excluded participants with no imaging or genetic data. To ensure HV models represented the spectrum of healthy aging, participants were excluded based on history of impairment (neurological or psychiatric disorders, head trauma, substance abuse, or cardiovascular disorders). Finally, to control for population level genetic heterogeneity, only participants with self-reported ‘British’ ethnic backgrounds were considered. Imaging and genetic protocols are described in [34] and [37], respectively. The dataset preparation followed the process described by [38], with HVs calculated using FreeSurfer V6 [39].

#### ADNI

The Alzheimer’s Disease Neuroimaging Initiative (ADNI) was launched in 2003 as a public–private partnership, led by Principal Investigator Michael W. Weiner, MD. The primary goal of ADNI has been to test whether serial magnetic resonance imaging (MRI), positron emission tomography (PET), other biological markers, and clinical and neuropsychological assessment can be combined to measure the progression of mild cognitive impairment (MCI) and early Alzheimer's disease (AD). Genetic and imaging protocols are described in [40, 41] respectively. From a total of 2,430 (1,157 female) participants available in the ADNI dataset (downloaded Sep 2024), aged 50–91, we included the subjects with FreeSurfer v7 imaging/HV available as described in [42]. Furthermore, we used genotyping data of ADNI participants pre-processed as previously described by (Scelsi et al., 2018) [43]. We filtered out subjects with < 0.8 predicted European ancestry based on SNPWeights Genetic Principal Components (GPCs) [44].

#### EPAD

The European Prevention of Alzheimer’s Disease study (EPAD) is a growing longitudinal study of over 2500 (mostly non-demented) participants over the age of 50 with imaging, genetic, and clinical data, collected from 31 sites across Europe (Ritchie et al., 2019) [36]. Data from a total of 2660 subjects (1457 female) aged 50–89 were obtained with HVs calculated using FreeSurfer V6. We filtered out subjects with < 0.8 predicted European ancestry based on SNPWeights Genetic Principal Components (GPCs).

### Polygenic scores

We built our polygenic scores using Clumping and Thresholding (C + T) with PRSice V2 [45]. We used a previously reported HV GWAS of 26,814 European subjects from the ENIGMA study [7]. We filtered for minor allele frequency of 0.05, genotype missingness of 0.1, and performed clumping at 250 Kb. We calculated PGSs at 13 P-value thresholds (1E-8, 1E-6, 1E-5, 1E-4, 1E-3, 0.01, 0.05, 0.1, 0.2, 0.4, 0.5, 0.75, 1) using the sum of effect sizes weighted by SNP allele count. Additional File 1: Table S2 provides an overview of loci involved in PGS scores using P < 1.e-5 with functional annotation using the Functional Mapping and Annotation (FUMA) tool [46] and SNP-to-gene mapping using positional and eQTL mapping with brain-specific datasets (GTEx v8 [47]). Next, we converted the PGSs to Z-scores and regressed out the effect of genetic principal components (GPCs) using linear regression. We used 40 GPCs previously calculated from a select independent set of SNPs from UKBB subjects using fastPCA [48].

To compute the double PGS, i.e., a PGS considering polygenic scores at two P-value cutoffs, their corresponding Z-scores are summed.

#### LASSO-PGS

We derived a LASSO-PGS by using weights from a LASSO regression to linearly combine the PGSs at all 13 thresholds after Z-scoring. Here the target variable in UKBB was bilateral HV and the inputs were the 13 PGSs at different P-value thresholds. We used k-fold cross-validation to tune the regularization parameter (λ). Across all experiments, LASSO weights were derived from a UKBB training set and applied to the testing sets (UKBB testing, ADNI and EPAD). To achieve robust statistics, LASSO training was bootstrapped 30 times.

#### Cross dataset common polygenic score

For experiments that were carried out across datasets, it was required to harmonize the PGS. First, we only considered SNPs that were available in all three databases for the PGS computation (genotyped UKBB, imputed ADNI and EPAD). Next, we estimated genetic ancestry using the SNPWeights tool [44], which is based on a large external reference panel. We used NeuroCombat [49, 50] to harmonize the PGS values to UKBB by retaining the effect of the SNPWeights GPCs. After the harmonization step, we linear regressed out the effect of the GPCs on PGSs across all three datasets.

### Hippocampal volume

For HV and ICV, we computed bilateral HVs from left and right HVs generated by FreeSurfer [39] and we used estimated total intracranial volume (eTIV) as our ICV measurement. HVs were generated using FreeSurfer v6 for EPAD and UKBB and FreeSurfer v7 for ADNI.

#### Cross dataset harmonization

Similarly to the harmonization of the PGS values, also the imaging biomarkers must be harmonized to account for biases induced by study protocols and processing pipelines. We used NeuroCombat [49, 50] to harmonize the HV and eTIV values to the UKBB. This was conducted in two steps: first eTIV was harmonized retaining the effects of age, sex and GPCs. Second, we harmonized the HVs retaining the effects of age, sex, diagnosis, harmonized eTIV and PGS.

#### PGS correlations

We computed the Pearson correlations of the 13 scores with each other and with HV. Furthermore, we investigated the ranking of subjects by PGS in two ways. Firstly, we directly examined how the PGS-based ordering of subjects changed across PGSs. Secondly, we computed the overlap between the top/bottom 30% of subjects sorted by each of the 13 PGSs.

### Estimating nomogram separation

For each PGS (single/double/LASSO), we built nomograms from the top vs bottom 30% scoring participants using Gaussian Process Regression (GPR). The method follows our previously described work [10]. Briefly, our GPR models were trained with the laGP [51] R library, with the commonly used squared exponential covariance kernel function. After training, we build nomograms by predicting HVs within the training age range with increments of 0.01 years and calculating the quantile curves at nine percentiles: 2.5%, 5%, 10%, 25%, 50%, 75%, 90%, 95%, and 97.5%.

To derive the difference between two nomograms, we computed the average difference between their corresponding quantile curves. To this end, we first restricted the range of the nomograms to the intersection of their training data age range, because GPRs tend to diverge quickly where no training data is present rendering comparisons outside this range unreliable. Next, we calculated the difference between two nomograms across the age range at their matching percentile curves (e.g., at 2.5%). The nomogram difference is then defined as the average difference between all pairs of quantile curves.

### Multivariable normative model

#### Model training

In our previous work [10], stratification led to nomogram separation when comparing top/bottom strata, but when the middle strata was included (top vs bottom 50%) nomogram separation is greatly diminished. Hence, we can make better use of the data by building higher dimensional GPR models instead. Higher dimensional models are capable of learning from all the input covariates (age, ICV, PGS, etc.) jointly and can still be a basis of nomograms for different strata of covariates. The extracted z-score is the key information to be extracted from normative models, which can be done with GPRs of any dimension.

We trained GPRs with 17 progressively complex sets of covariates. The models produce HV z-scores given only Age (A Model), and Sex (AS Model), and ICV (ASI Model), and PGS (ASIP Model), where PGS is either the LASSO-PGS (ASIP-LASSO) or one of 13 single threshold PGSs (ASIP-single: ASIP-1E-8 to ASIP-1). To achieve robust LASSO models, the UKBB was randomly split in half 30 times and both LASSO-PGS and ASIP-LASSO were generated each time. For model assessment, we report the root mean squared error (RMSE) and the Pearson’s correlation coefficient of the z-scores to HV in the training, testing, and out-of-sample tests.

Importantly, we limit our testing to subjects whose ages and ICVs are within the range seen by the trained model, since GPR diverges quickly when extrapolating outside the range of training values.

#### Neurocognitive tests

We tested the association between improved normative models in out-of-sample data on a range of neurocognitive measures depending on their availability in the corresponding dataset. For example, both ADNI and EPAD had Mini-Mental-State-Examination (MMSE) and Clinical Dementia Rating Sum of Boxes (CDR-SB), while ADNI also provides the Alzheimer's Disease Assessment Scale (ADAS), and EPAD had the Repeatable Battery for the Assessment of Neuropsychological Status (RBANS).

We report the significant p-values after Holm–Bonferroni correction across five tests in each dataset independently. We also looked at the performance across five domains (Memory, Language, Orientation, Visual-Spatial, Executive Function) by combining subtests of NC measures where available. For example, in ADNI, the Memory domain consisted of: Everyday Cognition – study partner memory score, MMSE recall task, ADAS memory task, and the MOCA memory task. The details of which tests were used from each database, and which sub-tests composed every domain can be found in Additional File 1: Table S3—S5. Tests were conducted for each of the sub-tests independently and then combined using Stouffer’s method adjusted for dependent P-values (implemented in the *poolr* R library [52]) to provide a domain level *P*-value.

#### Defining Model-Shift

The aim is to quantify the improved association between NC values and GPR normative models constructed using the LASSO-PGS compared to classic normative models. In order to compare the z-scores from two GPR models, we consider the difference between them in terms of the subjects’ predicted percentiles. Specifically, we look at how much the percentile shifts when going from ASIP-LASSO to any other model (e.g., ASI), a value we term *model-shift*. If a subject’s percentile in the ASIP-LASSO model is higher than in the other model, their model-shift would be positive, e.g., + 5%, and vice versa. Thus, positive model-shifts suggest that the ASIP-LASSO model rates the HV value more normal. Thus, we expect positive model-shifts to represent better neurocognitive function such as better memory.

#### Cross sectional analysis

We used linear regression to assess the effect of model-shift (in percentiles) on the cognitive measures. These models were adjusted for standard covariates such as age, sex, ICV and the percentile from a GPR model, which ASIP-LASSO was compared to. We also conducted two sensitivity analyses: (i) to ensure that our results were not driven by subjects with high model-shifts, we performed the same tests while excluding subjects where model-shift magnitude was greater than 10% and (ii) in ADNI, to ensure our results were not driven completely by people with dementia, we also performed the tests after excluding participants diagnosed with Dementia at the baseline visit.

#### Longitudinal analysis

Leveraging the longitudinal design of the ADNI and EPAD studies, we used linear mixed effects models to assess whether the model-shift had longitudinal impact. More precisely, we estimated three sets of models. One with age, sex, ICV, baseline percentile, visit-time, baseline percentile and time interaction as fixed effects and subject ID and visit-time as random effects (termed ‘*MS-none*’ since model-shift is not included). A second model that adds model-shift as a fixed effect on top of MS-none (*MS-fixed*), and a third that adds the model-shift and time interaction as fixed effects on top of MS-fixed (*MS-time*). We then report the results of an ANOVA test between MS-none versus MS-fixed, and MS-fixed versus MS-time. We performed this test with ASIP-LASSO versus ASI model-shift, and ASIP-LASSO versus each of the 13 single threshold ASIP-PGS model-shift. In order to illustrate the longitudinal results, we plotted, across time, the predicted performance with the mixed effects models for an average participant with + 10% model-shift versus −10% model-shift. Similar to the cross-sectional tests, we conducted sensitivity analyses by excluding outliers in terms of |model- shift|≥ 10% and excluding Alzheimer’s Disease subjects from ADNI.

#### Crossing label longitudinal analysis

We used linear mixed effects models to assess whether crossing a specific percentile curve between the ASI and the ASIP-LASSO models had any longitudinal impact. For each percentile (1–100), we defined a percentile *crossing-label* depending on whether the model-shift from ASI to ASIP-LASSO did not cross that percentile (0), crossed above it (−1), or crossed below it (+ 1). We then constructed linear mixed effects models with age, sex, ICV, baseline Z-score, crossing-label, visit-time, crossing-label and time interaction as fixed effects, and subject ID and visit-time as random effects. We report p-values that, in addition to being corrected for five NC tests and five domains in each dataset, are also corrected for the 100 percentiles tested using FDR Correction.

#### Time to decline analysis

Finally, we used Cox Proportional Hazard models to assess the impact of model-shift on the length of time for CDR-SB decline. As suggested by Andrews et al. [53], a CDR-SB increase of 1.0 to 2.0 points is considered a minimally clinically important difference (MCID) in groups with mild to moderate dementia. In ADNI participants with baseline CDR-SB of 1.0 we conducted two survival analyses for the time to reach a 1.0 and a 2.0 increase in CDR-SB, respectively. The model covariates were baseline age, sex, APOE-e4 count, normative percentile and model-shift.

## Results

### PGS at different thresholds contain complementary information

We built normative models using data from UKBB participants. In total, we included data from 23,997 participants, after applying exclusion criteria for existing health conditions and genetic ancestry (Table 1). To measure the impact of the genetic information, participants were stratified into two groups: genetically predicted larger and smaller hippocampi. Following previous work by Janahi et al. [10], separate nomograms were estimated for both strata and the degree of separation was computed (Methods). PGS were generated using PRSice-2 [45] on harmonized SNP data (Methods). The most lenient (P = 1) and most stringent (P = 1E-08) P-value cutoff comprised 70,251 and 4 SNPs, respectively. The most significant 40 SNPs were found to be associated with the *LEMD3*, *TESC*, and *MSRB3* genes among others (Additional File 1: Table S2).

#### Single threshold PGS correlations

**Fig. 2:**
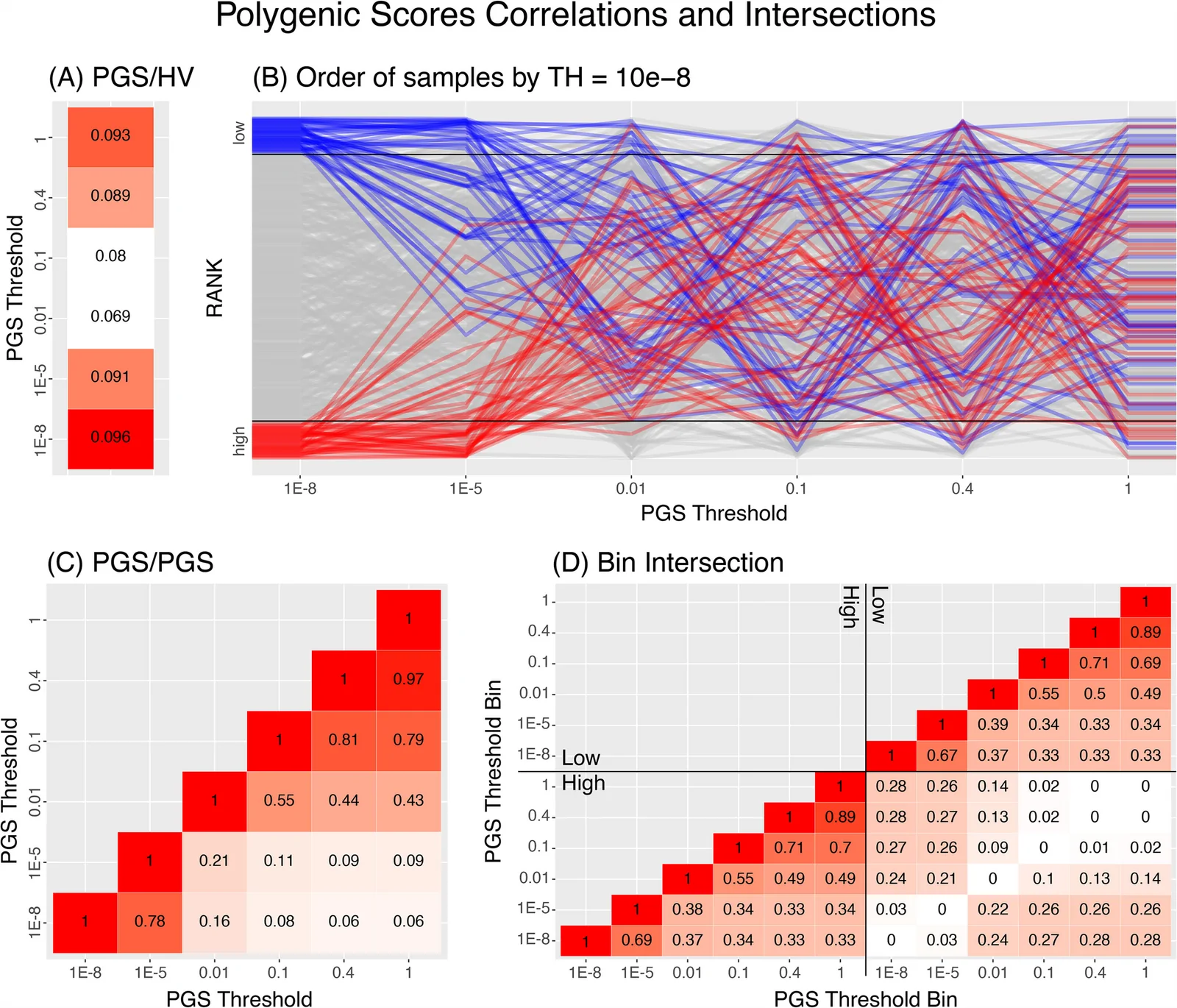
HV PGS Correlations in UKBB. **A** HV PGSs generated at different P-value thresholds correlate similarly to HV (*P* = 1E-8 & *P* = 1 exhibit Pearsons *r* = 0.09). **B** The order of participants is not preserved when ranking them by the *P* = 1E-8 threshold PGS. The top and bottom 10% of participants (blue/red lines) are essentially randomly distributed among the ranks of participants by the *P* = 1 threshold PGS. **C** These same PGSs become less correlated as the distance between the thresholds increases; ranging from r = 0.97 down to r = 0.06. **D** Overlap of participants ranked in the top/bottom 30% by each PGS. Participants ranked high/low by two polygenic scores (bottom-left/top-right) overlap more than those ranked high by one score, but low by the other (bottom-right). For instance, 33% of the participants ranked as high at the *P* = 1E-8 threshold are also ranked high at the *P* = 1 threshold. However, 28% of the participants ranked high at the *P* = 1E-8 threshold are also ranked low at the *P* = 1 threshold. The diagonal of the bottom right sector of the table is all zeros since it represents the intersections of the top and bottom bins in the same threshold PGS

HV PGSs at different thresholds exhibited consistent correlations with measured HV, with correlations being strongest at the opposing ends (Fig. 2 A, Additional File 2: Fig S1). PGSs using similar P-value thresholds show strong correlations between each other, while PGSs using distant P-value thresholds appear uncorrelated (e.g., P = 1E-8 and P = 1.0 exhibit Pearson’s *r* = 0.06) (Fig. 2 C, Additional File 2: Fig S2).

When considering an integration of PGS into normative models, it is important to consider the ranking the PGS generates for the participants. Indeed, when subjects are ranked with respect to a PGS using P-value threshold 1E-8, then they appear nearly randomly distributed when viewed at the *P*-value threshold of *P* = 1.0 (Fig. 2B, Additional File 2: Fig S3). That is, PGSs at different thresholds split participants differently. For instance, of the subjects ranked high according to the *P* = 1E-8 threshold (i.e., highest 30% of subjects by HV PGS), only 33% were also ranked high using the *P* = 1 threshold, while 28% were ranked low at the *P* = 1 threshold and the remaining 39% were ranked in the middle (Fig. 2D; Additional File 2: Figs S2/S4). As before, overlap between groups was higher when the two *P*-value cutoffs were closer.

#### LASSO-PGS

These previous analyses showed that the HV-PGSs constructed at different thresholds carry complementary information. Next, we used a LASSO regression [54] to combine the scores at different cutoffs into a single score (LASSO-PGS), which we hypothesize to better correlate with HV than single scores (Methods). To this end, we trained 30 bootstrap replicates of sex-stratified models on UKBB. The LASSO model consistently put higher weights on the scores with the highest and lowest thresholds (Additional File 2: Fig S5).

### Combining PGSs improves nomogram separation

**Fig. 3:**
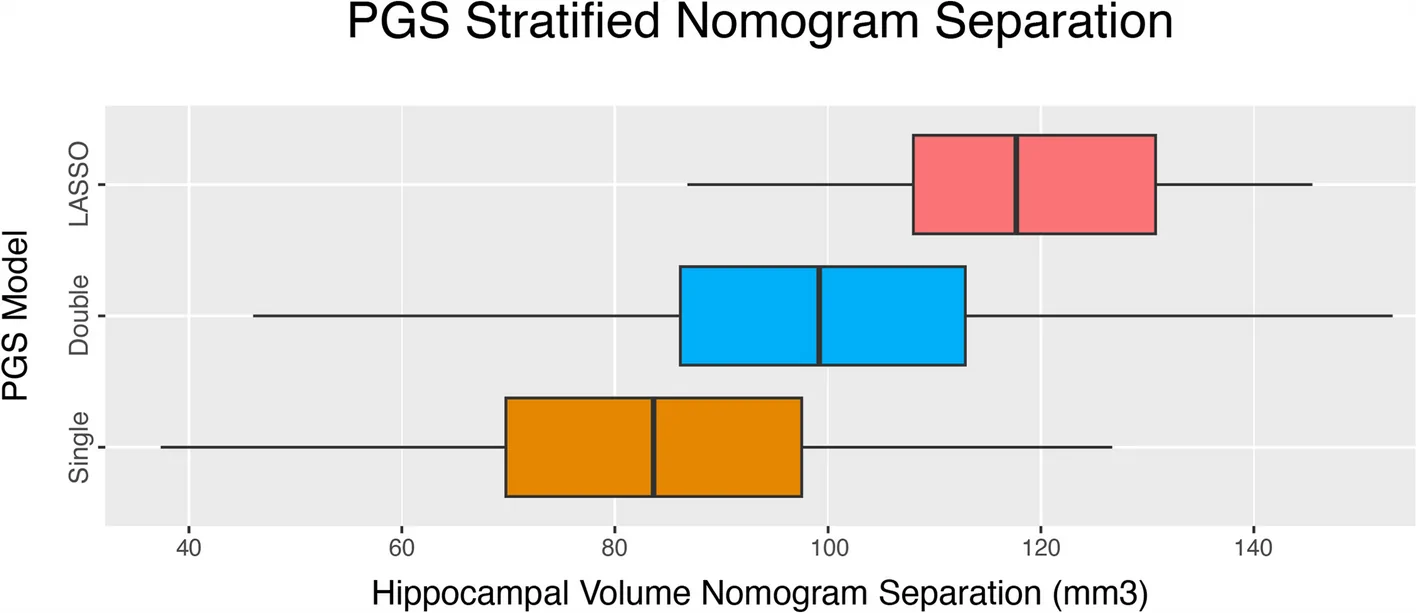
Combining multiple PGS improves Nomogram Separation. Comparing the nomogram separation in UKBB across 30 bootstraps of stratification by 13 single threshold PGSs of HV; 78 combinations of two thresholds; and a lasso of all 13 thresholds. On average, a double-PGS is 22% better than a (classic) single threshold PGS, and a LASSO-PGS is 50% better

To evaluate the effect of the different PGS on the normative models, we constructed different nomograms for the subjects ranked in the highest and lowest 30% according to the PGS. Next, we computed the level of nomogram separation between the “high” and the “low” groups (Methods). This was conducted for the LASSO-PGS and as a reference for all single PGS thresholds and all combinations of two thresholds (double-PGS). Compared to a single PGS threshold, using the double- or LASSO-PGS improved nomogram separation. On average, double threshold PGSs were 22% better than single threshold PGS, and that the LASSO-PGS was 50% better (Fig. 3, Additional File 2: Fig S6/S7).

### Combining PGSs improves HV models in out-of-sample datasets

After having established that combining multiple PGS using a LASSO regression provides a better separation between nomogram strata, we aimed to build multivariate normative models that can be applied to external datasets to improve diagnosis and prognosis of people with dementia. After applying inclusion criteria based on genetic and imaging data availability and matching genetic ancestry, 23,997 participants were included from UKBB for training, and for evaluation 1,245 participants (6,814 visits) from ADNI and 1,755 participants (4,938 visits) from EPAD (Table 1).

#### Harmonization and cross dataset common polygenic score

To have comparable PGS across datasets, the SNP content across all three databases was harmonized (resulting in 262,660 common SNPs; Methods) and PGS were recomputed at the 13 cutoffs with the most lenient cutoff (*P* = 1.0) comprising 53,686 SNPs. Dataset specific biases were removed prior to further downstream analysis (Methods; Additional File 2: Fig S8).

#### Model training

**Fig. 4:**
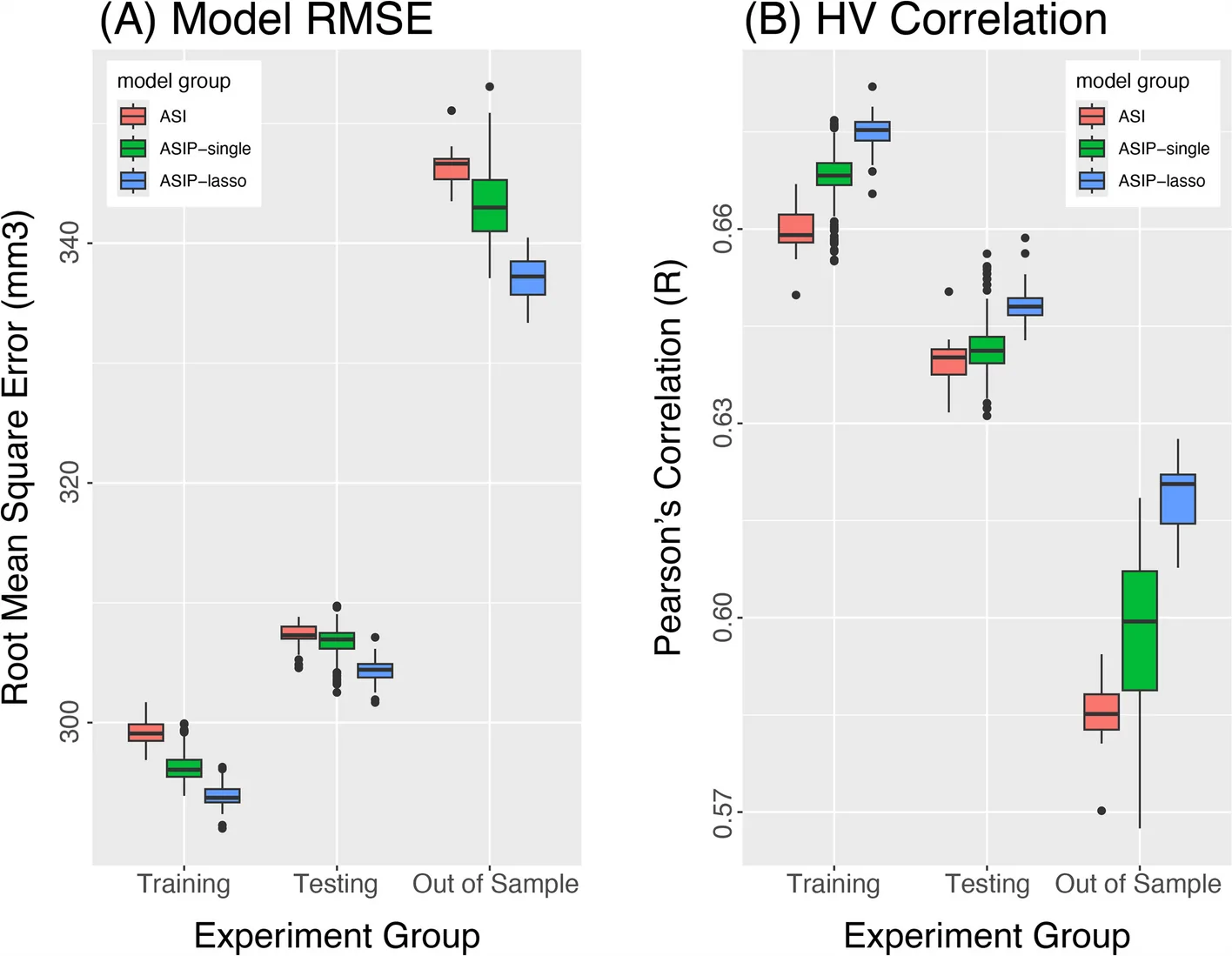
High Dimensional GPR models across datasets and covariates. **A** Root Mean Squared Error (RMSE) between predicted HV by the GPR models and measured HV. On average, the RMSE lowers when going from the standard Age-Sex-Intracranial (ASI) model (red) to the ASI + polygenic (ASIP) using single threshold PGS (ASIP-single; green), to the ASIP-LASSO model (blue). RMSE increases on test data and out-of-sample cohorts. **B** Pearson’s correlation coefficient between predicted and measured HV by the different models

We randomly split UKBB into training (50%) and testing (50%) cohorts and trained different normative models using Gaussian Process Regression (GPR). For all models, HV was the target variable, and all models contained age (A), sex (S) and intracranial volume (I) as predictors (“ASI” models). In addition, we trained models that used a single PGS as predictor (ASIP-single) and models that used the LASSO-PGS as predictor (ASIP-LASSO). The trained models were applied to the UKBB test cohort, and to both ADNI and EPAD participants. We found that GPR models improved when incorporating genetic information, with the strongest improvement being seen with the LASSO-PGS (Fig. 4). Unsurprisingly, RMSE increased when going from training to testing in UKBB and further when applying the models to out-of-sample datasets. When evaluating the model performance by diagnosis, EPAD and CN participants in ADNI exhibited similar performance compared to participants in the UKBB test set (Additional File 2: Fig S9/S10). This indicates that larger deviations in performance are caused by disease effects, demonstrating the underlying assumption of normative models. Overall, the relative performance of all tested strata improved when adding single threshold PGS and further with LASSO-PGS.

### Combining PGSs improves prediction of cognitive performance cross-sectionally and longitudinally

**Fig. 5:**
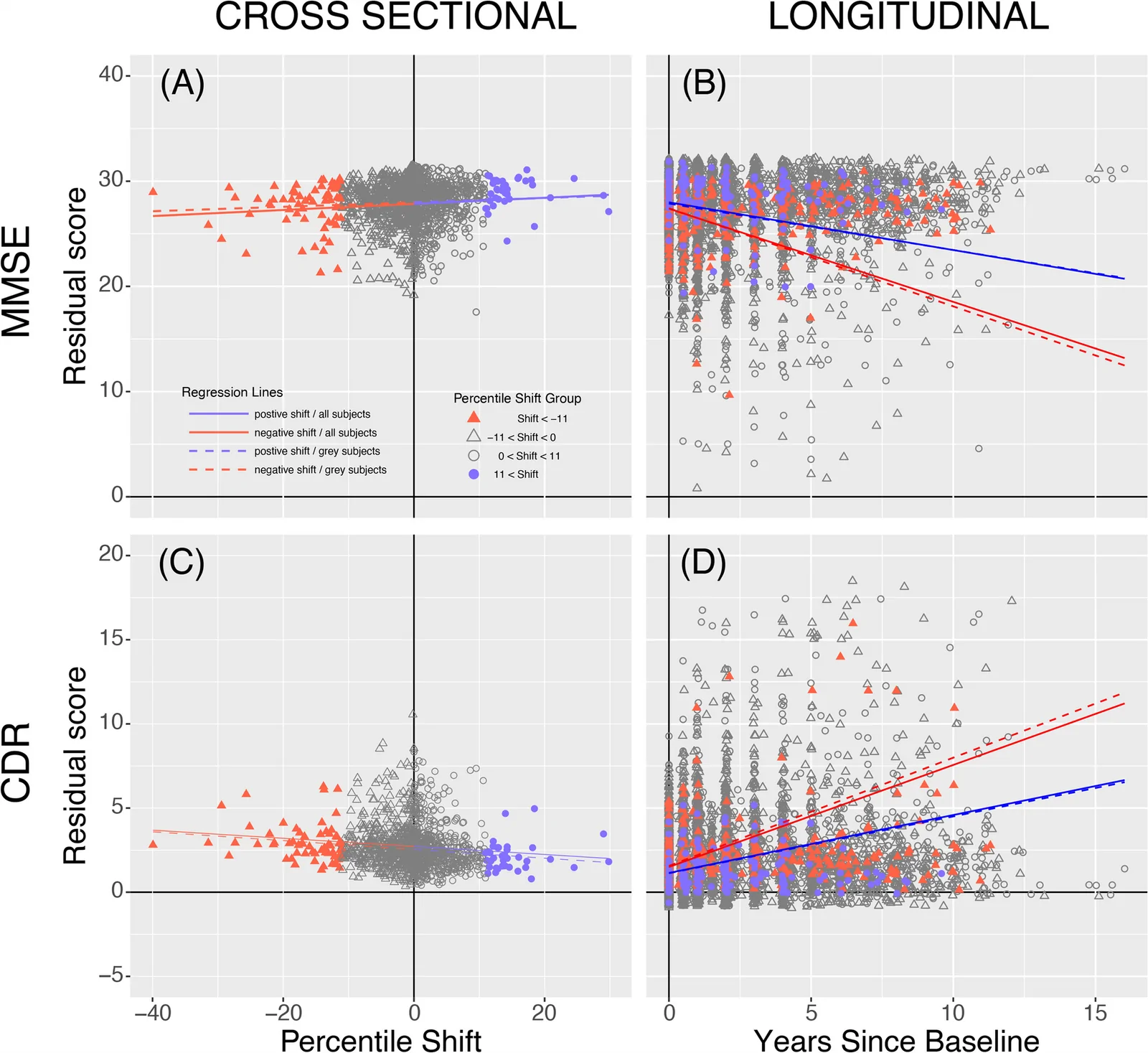
Cross-sectional and Longitudinal Effect of Model-shift. Illustrative example of comparing performance in MMSE and CDR-SB in ADNI to ASIP-LASSO to ASI model-shift. The y-axis represents the residual scores after removing the effects of age, sex. (**A**, **C**) At baseline, model-shift is positively correlated to MMSE (*P* = 2.6E-3), where a higher score is better; and negatively correlated to CDR (*P* = 7.7E-5), where a lower score is better. When observing moderately shifted participants (|model-shift|< 11%; grey points) the CDR-SB correlation remains significant (*P* = 3.6E-3) and the magnitude are not significantly changed (dashed line). (**B**, **D**) These correlations remain significant longitudinally (MMSE: *P* = 3.6E-4, CDR-SB: *P* = 8.4E-4). The regression lines drawn are projections/prediction for an average subject with + 10 vs −10 model-shift (blue vs red)

In a final set of experiments, we challenged the clinical utility of these genetically adjusted normative models. We used the rich phenotyping data available in both ADNI and EPAD to assess whether adding genetics into the normative models improves the association with current and future cognitive function. In these experiments we computed participants’ percentile score using the ASI model as well as the ASIP-LASSO model. Next, using linear models and linear mixed effect models we tested whether the percentile shift induced by the ASIP-LASSO model compared to the ASI model (termed *model-shift*) better explains current and future test scores, respectively (Methods). Cross-sectionally, we found significant correlations to multiple neurocognitive (NC) tests and domains across both out-of-sample datasets. In both ADNI and EPAD, the model-shift introduced by the LASSO-PGS demonstrated significant added benefit over the classic normative model (ASI) for MMSE and CDR-SB (Fig. 5, Additional File 2: Fig S11/S12, Table 2, Additional File 1: Table S3). In ADNI, ASIP-LASSO significantly improved the association with ADAS13 and MOCA. When limiting the analysis to participants showing moderate model-shift (< 11%), there was significant association with CDR-SB and Flanker in ADNI and EPAD, respectively. Results were similar when comparing ASIP-LASSO to ASIP-single; even after excluding participants with dementia from ADNI (Additional File 1: Table S3). Model-shift was significantly associated to all cognitive domains in ADNI. These associations remained significant when only participants with moderate shift (< 11%) were considered, i.e., these results were not driven by few extreme cases with strong model-shift. Notably, the magnitude of the *model-shift* did not show any bias regarding diagnostic groups in ADNI (Additional File 2: Fig S13). Moreover, when stratifying the ADNI cohort by APOE-e4 genotype, associations remained significant within the strata (Additional File 1: Table S7), indicating that our results were not driven by APOE-e4 genotype contributing to some HV PGS. In EPAD, the model-shift was significantly associated with Memory and Executive Function (Table 2).

**Table 2:** Neurocognitive test/domain association with model-shift

TABLE	NC Test/Domain	Cross Sectional Results				Longitudinal Results			
		All		No High Shift		All		No High Shift	
		slope	p-val	slope	p-val	slope	p-val	slope	p-val
ADNI	MMSE	.029	**2.6E-03**	.021	1.3E-1	.022	**3.6E-04**	.025	**5.4E-03**
	CDR-SB	-.024	**7.7E-05**	-.026	**3.6E-3**	-.013	**8.4E-04**	-.015	7.5E-03
	ECOG.SP	-.008	1.0E-02	-.008	7.6E-2	-.0009	2.1E-01	-.001	3.3E-01
	ADAS13	-.149	**2.1E-05**	-.129	1.1E-2	-.033	1.1E-02	-.044	2.2E-02
	MOCA	.053	**5.5E-03**	.059	2.6E-2	.012	1.2E-02	.013	7.4E-02
	Memory		**5.8E-10**		**9.4E-04**		2.4E-01		1.6E-01
	Language		**1.8E-03**		**9.4E-04**		**5.9E-03**		3.5E-02
	Orientation		**1.4E-04**		**2.1E-02**		**4.5E-06**		**5.5E-04**
	Visuospatial		**1.5E-02**		**2.1E-02**		1.5E-01		7.0E-01
	Exec. Function		**2.5E-05**		**3.4E-03**		**1.4E-03**		8.0E-02
EPAD	MMSE	.019	**3.0E-03**	.014	1.6E-1	.004	8.8E-01	.002	8.0E-01
	CDR—SB	-.007	**9.2E-03**	-.008	3.8E-2	-.0005	8.5E-01	-.001	4.7E-01
	RBANS	.112	3.9E-02	.139	9.2E-2	-.005	6.1E-01	.025	5.7E-01
	Flanker	.007	5.8E-02	.015	**6.5E-3**	-.002	1.3E-01	-.003	2.5E-01
	Dot Count	.033	1.0E-01	-.005	8.7E-1	-.002	1.2E-01	.035	1.7E-01
	Memory		**9.6E-05**		**4.0E-03**		8.9E-01		9.4E-01
	Language		6.9E-01		4.3E-01		4.8E-01		2.6E-01
	Orientation		1.8E-01		2.5E-01		1.9E-01		4.3E-01
	Visuospatial		8.1E-02		2.6E-01		5.7E-01		1.5E-01
	Exec. Function		**4.0E-05**		5.3E-02		1.7E-01		3.4E-01

Bold items: statistically significant after Holm–Bonferroni correction across 5 sets of tests/domains in each dataset independently. Correction was done independently in cross-sectional/longitudinal, and all/no-high-shift groups. Cross sectional P-values are derived from linear models, and longitudinal p-values are derived from an ANOVA between two Linear Mixed Effect (LME) models (see Methods). *MMSE* Mini-Mental State Examination, *CDR-SB* Clinical Dementia Rating Sum of Boxes, *ECOG SP* Everyday Cognition Study Partner, *ADAS13* AD Assessment Scale13 items, *MOCA* Montreal Cognitive Assessment, *RBANS* Repeatable Battery for the Assessment of Neuropsychological Status

In our longitudinal linear mixed effects models, model-shift was significantly associated with MMSE and CDR-SB in ADNI (Fig. 5, Table 2, Additional File 1: Table S3), with shifts towards larger percentiles indicating a longitudinally better cognitive profile. In the domain tests, we found significant longitudinal association with Language, Orientation, and Executive Function (P = 5.9E-3, P = 1.5E-5, P = 1.4E-3, respectively) (Table 2). There were no significant longitudinal associations in the EPAD cohort. Likely owing to the short-term follow-up data compared to ADNI. Similar to our cross-sectional results, APOE-e4 stratification did not results in significant differential results across the strata (Additional File 1: Table S7).

Percentile shifts are not uniformly meaningful; crossing the 50th percentile may have less significance than crossing the 5th percentile for example. To test whether this was the case in our models, we recorded whether the ASI to ASIP-LASSO *model-shift* crossed predefined percentile thresholds and tested whether these crossings were associated with longitudinal performance using linear mixed-effects models (Methods). In ADNI, crossing at the 1 st and 10th percentile thresholds was significantly associated with performance in multiple neurocognitive tests and all domains (Additional File 2: Fig S14/S15, Additional File 1: Table S3/S6). For example, MMSE and CDR-SB showed significant effects at the 1 st percentile (P = 4.3E-15 and P = 2.1E-9) and the 10th percentile (P = 4.5E-7 and P = 2.3E-7).

Finally, *model-shift* made a significant impact on the time it took participants with mild to moderate dementia to reach a MCID in CDR-SB. Of 591 eligible ADNI participants with CDR-SB of 1.0 or larger at baseline (MCI = 448; AD = 143), there were 317 and 234 progressions at CDR-SB increase of 1.0 and 2.0, respectively. Model-shift showed a statistically significant contribution at both thresholds (log HR = 0.076; P = 0.015 at 1.0; log HR = 0.039; P = 0.00895 at 2.0) in the expected direction.

## Discussion

In this study, we postulate a novel approach for constructing PGS at different thresholds to capture complementary genetic signals and integrate that to enhance predictive performance. We tested this approach using hippocampal volume (HV)—a key biomarker of Alzheimer’s disease—as a case study. While individual threshold-based PGSs correlated with HV, their inter-correlations varied substantially (R = 0.06 to 0.97) and participant rankings often diverged, confirming prior evidence that PGSs at different thresholds capture distinct genetic signals [33, 55, 56]. By harnessing this heterogeneity through a LASSO-based combination of 13 thresholds, we developed more robust and scalable models that consistently outperformed single-threshold or no-PGS models across multiple metrics. In particular, our LASSO-PGS models increased separation between high and low genetic risk strata from ~ 82 mm3 (using single-threshold PGSs) to ~ 118 mm3. Among healthy older adults [57], this improvement equates to a 2.4-year difference in age-related hippocampal atrophy -compared to just 1.5 years with single-threshold models – highlighting the clinical relevance of incorporating multi-threshold genetic information into brain health modelling.

Machine learning and precision diagnostic approaches that integrate genetics and medical imaging face common challenges when implemented in clinical settings.

A major challenge is ensuring that trained models and biomarkers maintain robust performance despite distribution shifts between training and application setting, whether due to differences in input data, acquisition protocols or processing pipelines [58]. This challenge also applies to normative models, especially those combining multimodal data. Such shifts can stem from variations in technical parameters, including MR scanner hardware, pulse sequences and genotyping platforms. To address this, we harmonized the SNP content prior to PGS generation to ensure comparability of scores across datasets. We further harmonized the generated PGS and imaging biomarkers using ComBat harmonization [49] to align feature distributions. Our evaluations revealed that performance (i.e., prediction errors and correlations) declined when models trained on UKBB were applied to independent samples in ADNI and EPAD—reflecting real world distribution shifts. Notably, this decline was primarily driven by people with cognitive decline in ADNI. When evaluated separately, performance in EPAD, which comprises participants at risk of dementia, was comparable to the UKBB test set. Moreover, within ADNI, the model performed better for cognitively normal participants, but worse for those with MCI or dementia, likely due to greater diagnostic precision in in ADNI compared to UKBB which may include undiagnosed cases. These results underline that our harmonization steps produced models that generalize reliably to novel data sets and that reduced performance in diagnosed cases reflect the intended functioning of the normative models.

Previous studies have demonstrated that hippocampal volume is a strong biomarker for Alzheimer’s disease progression [59, 60], commonly used as a primary or secondary endpoint in clinical trials [61–64] and correlates well with neurocognitive scores [65–67]. Our results build on this by demonstrating augmenting normative models with genetic information enhances their association with neurocognitive test performance. Specifically, we introduced *model-shift*, the change in an individual’s assigned percentile when PGS are included versus a conventional demographic-only model (ASI). This enabled us to quantify the added value of incorporating genetic information directly. Across both out-of-sample cohorts, *model-shift* was significantly associated with MMSE and CDR-SB scores both cross sectionally and longitudinally, outperforming models without genetics and those using single threshold PGS. Importantly, these associations were not driven by a small subset of individuals with large *model-shifts* (> 10%); even subtle adjustments improved the alignment with neurocognitive performance. *Model-shift* did not differentially impact diagnostic groups in ADNI overall; however, participants with dementia typically had shift values due to severe HV reductions yielding low baseline percentile scores that remained unchanged after genetic adjustment. When test sub-scores were grouped by cognitive domain, memory and executive function – domains closely linked to the hippocampus [68–72] – showed the strongest cross-sectional effects.

Beyond baseline predictions, the genetically adjusted normative model also improved the prediction of longitudinal cognitive trajectory. For example, MMSE and CDR-SB in ADNI demonstrated a positive model-shift-by-time interaction, indicating that individuals with greater model-shift showed a slower decline over time (Table 2). Notably, the orientation – strongly linked to hippocampal function [73, 74] – was most influenced longitudinally. In ADNI, participants falling below the 10th percentile using the genetically improved normative model exhibited differences in CDR-SB (0.57) and ADAS13 (1.74) over 18 months, comparable to the effect of Lecanemab in a clinical trial over the same period [75]. Consistent with these findings, survival analyses demonstrated that model-shift was also associated with time to clinically meaningful decline. Specifically, higher model-shift (i.e., participants showing less HV loss) was linked to a reduced hazard of reaching both 1-point (logHR = 0.076, p = 0.015) and 2-point (logHR = 0.039, p = 0.0089) increases in CDR-SB, indicating a slower progression to clinically meaningful worsening.

There are some limitations to consider in our study. Importantly, the datasets used differed in MR hardware, pulse sequences, processing software and genotyping arrays. Although we applied widely used harmonization techniques (such as imputation [76] and NeuroComBat [49]), ideally acquisition and processing parameters should be closely matched to minimise confounding variance – though our approach mimics real world scenarios. Another limitation is that our focus on hippocampal volume, despite its relevance to several disorders [77], comes with measurement challenges due to its shape and location. Although test–retest reliability has improved with newer FreeSurfer versions [78], HV remains among the least reliable subcortical volume estimates [79]. Using a dedicated pipeline for hippocampus segmentation could yield even more accurate measurements, further strengthening model performance. Additionally, the UKBB training cohort may contain undiagnosed cases and does not cover participants beyond age 82, limiting evaluation in older populations within ADNI and EPAD. To address these gaps, future models may benefit from increased size and diversity of training datasets and excluding participants based on AD diagnosis proxy measures.

## Conclusions

In conclusion, our study demonstrates that polygenic scores constructed at multiple thresholds capture complementary genetic signals that can be leveraged to build more predictive models of HV. By integrating these genetic predispositions directly into normative models, we show how medical imaging data and genetics can be combined to improve predictions of neurocognitive outcomes in independent datasets. This approach exemplifies how multi-modal data integration can advance precision medicine and holds promise for expanding PGS applications beyond neuroimaging.

## Supplementary Information

Additional file 1.

Additional file 2.

## Data Availability

The scripts and code used in this study have been made publicly available and can be found at Github (https://github.com/Mo-Janahi/Multi-Threshold-PGS) [80]. All underlying data, and derived quantities, are available by application from the UK Biobank (application number 65299) at http://www.ukbiobank.ac.uk, ADNI at http://adni.loni.usc.edu/data-samples/access-data/, and EPAD as https://ep-ad.org/index.php/open-source-data/. Summary statistics from the genome-wide association studies described in this paper is available from the NHGRI-EBI GWAS catalogue, study number GCST003961. URL: https://www.ebi.ac.uk/gwas/studies/

## Abbreviations

AD: Alzheimer’s Disease
ADAS: Alzheimer’s Disease Assessment Scale
ADNI: Alzheimer’s Disease Neuroimaging Initiative
APC: Article Processing Charge
APOE: Apolipoprotein E
ASI: Age, Sex, Intracranial Volume model
ASIP: Age, Sex, Intracranial Volume, Polygenic Score model
ASIP-LASSO: ASIP model including the LASSO-derived Polygenic Score
ASIP-single: ASIP model using a single-threshold Polygenic Score
C + T: Clumping and Thresholding
CDR: Clinical Dementia Rating
CDR-SB: Clinical Dementia Rating – Sum of Boxes
CN: Cognitively Normal
EPAD: European Prevention of Alzheimer’s Disease
eTIV: Estimated Total Intracranial Volume
FDR: False Discovery Rate
GPC: Genetic Principal Components
GPR: Gaussian Process Regression
GWAS: Genome-Wide Association Study
HV: Hippocampal Volume
ICV: Intracranial Volume
LD: Linkage Disequilibrium
LASSO: Least Absolute Shrinkage and Selection Operator
LME: Linear Mixed Effects (model)
MCI: Mild Cognitive Impairment
MMSE: Mini-Mental State Examination
MOCA: Montreal Cognitive Assessment
MRI: Magnetic Resonance Imaging
NC: Neurocognitive
PGS: Polygenic Score
PRS: Polygenic Risk Score
QC: Quality Control
RMSE: Root Mean Squared Error
SNP: Single Nucleotide Polymorphism
UKBB: UK Biobank
